# Strong feelings, small bites: investigating emotional undereating in children's picky eating and food neophobia within the family context

**DOI:** 10.3389/frcha.2026.1730322

**Published:** 2026-07-29

**Authors:** Juliette Taquet, Lien Goossens, Ellen Moens, Sandra Verbeken

**Affiliations:** 1 Department of Developmental, Personality and Social Psychology, Ghent University, Ghent, Belgium; 2 Odisee University College, Brussels, Belgium

**Keywords:** emotion regulation, emotional undereating, food neophobia, parenting style, picky eating

## Abstract

**Objective:**

Food neophobia and picky eating in young children are important barriers to developing healthy eating patterns. Given the crucial role that families, especially parents, play in shaping children's eating behavior, we aimed to examine emotional undereating underlying children's food neophobia and picky eating within the family context.

**Method:**

Parents (*N* = 170) of children aged 3–8 years (*M* = 5.45, *SD* = 1.69; 51.8% boys) reported on their child's food neophobia, picky eating, and emotional undereating. Parents also reported on their own food refusal behavior (modeling), pressure to eat (feeding practices), and emotional acceptance of the child (parenting style).

**Results:**

Mediation analyses showed that emotional undereating mediated the association between parental acceptance of the child and both food neophobia and picky eating. In contrast, parents' own food refusal behaviors and pressure to eat were directly associated with children's food refusal behavior. These associations were not mediated by emotional undereating.

**Discussion:**

The findings underscore the role of the parent-child relationship quality and emotion regulation in explaining children's food refusal behavior. Longitudinal research is needed to replicate and extend these results and further examine how family context variables and emotion regulation strategies contribute to the development and maintenance of children's food avoidance.

## 1 Introduction

Food neophobia and picky eating are important psychological barriers to increasing a child's dietary variety (1, 2). While food neophobia involves the reluctance to try unfamiliar foods, picky eating involves rejecting a wide range of familiar and unfamiliar foods, leading to a limited diet (3). Food neophobia and picky eating are closely related constructs (4), but are behaviorally distinct, with different factors predicting their severity and expression [e.g., (5, 6)].

Both food neophobia and picky eating tend to decrease with age (3, 6, 7), although their developmental trajectories differ. Food neophobia typically shows a gradual decline with food familiarity, whereas picky eating appears to be more persistent in a substantial subgroup of children (8–10). Food neophobia and picky eating have been linked to adverse outcomes, including poorer dietary quality (11) and increased symptoms of internalizing and externalizing psychopathology in children (12). In severe cases, some children may develop Avoidant/Restrictive Food Intake Disorder (ARFID) (13, 14). Additionally, child food neophobia and picky eating are associated with problematic parent-child feeding dynamics (15–17) and reduced parental wellbeing (12, 67).

Although food neophobia and picky eating have a heterogeneous etiology (3, 18, 19), a common factor is the distress often associated with food and eating, which may increase the likelihood of children avoiding food as a strategy to regulate these emotions. We refer to this pattern of responding as “emotional undereating”, defined as avoiding food in response to emotional states or arousal (20, 21). There is evidence for the robust link between eating disorders such as anorexia nervosa and emotion regulation difficulties (22, 23) and emotional undereating (24, 25), but it has been less studied in normative childhood eating behavior, with research mostly focusing on emotional overeating. However, these factors might also play a role in children displaying picky eating or food neophobia (26). Examining food neophobia and picky eating through an emotion regulation lens can enhance our comprehension of their development and persistence. While factors such as temperament, cognitive development and neurophysiology play a role in children's emotion regulation, the family context is also crucial to take into account. Recent reviews consistently highlight the central role of parents, feeding practices, and broader family functioning in the development and maintenance of children's eating behavior and feeding difficulties [e.g., (16, 27)]. The Tripartite Model of Familial Influence on Emotion Regulation (28) highlights three pathways through which the family context affects children's emotion regulation strategies, impacting their adjustment.

First, children learn to regulate their emotions through observation. In the context of food refusal, if parents model maladaptive strategies to regulate eating-related distress, such as avoidant eating behaviors to cope with distress, their children may adopt similar emotion regulation strategies. This could increase the likelihood of the child developing or maintaining avoidant eating behaviors. Research has shown that children learn to accept foods by observing significant others, and that parental food neophobia is linked to childhood food neophobia (29, 30). However, no studies have yet examined whether the association between parents' and children's food refusal is mediated by the child's emotion regulation difficulties.

The second pathway proposed by Morris et al. (28) posits that parents' reactions to their children's emotions and their perceived control over these emotions affects children's emotion regulation. Specifically, punitive or negative parental responses can heighten children's emotional arousal, leading them to avoid rather than understand and express their emotions appropriately (31). An optimal level of parental control appears to be needed, where children can express emotions, without being overly encouraged to do so (28). Mitchell et al. (32) reviewed evidence on how parental feeding practices contribute to feeding problems. Permissive and authoritarian approaches are related to increased feeding problems (33, 34). High parental control, such as pressuring a child to eat is associated with lower food intake and more negative reactions (e.g., (35, 36)). For instance, pressuring a child to consume novel foods can worsen their emotional state, leading to negative associations with the food (3). Conversely, permissive practices (e.g., preparing preferred dishes, offering limited variety, avoiding mealtime conflict) are also linked to increased distress and picky eating (32). These maladaptive feeding practices may exacerbate children's avoidant coping strategies, or emotional undereating, thereby increasing the risk of food refusal behavior.

The third pathway highlights how the emotional family climate, reflected in the quality of relationships, influences children's emotion regulation. A less sensitive and responsive parenting style can prevent children from developing a sense of security, leading to an avoidant coping style and increased risk of maladjustment (37). A recent longitudinal study found that children from less well-functioning families at age six were at higher risk of emotional undereating by age ten (20). Furthermore, research has shown that a poor family climate, marked by insecure attachment, is linked to problematic eating patterns such as emotional eating [e.g., (38)], as well as restricted eating behaviors in children and adolescents through avoidant emotion regulation strategies (39, 40). However, it remains unclear whether the association between the emotional family climate and food refusal behavior operates through emotional undereating as a maladaptive emotion regulation strategy.

In sum, although several parental factors have been consistently linked to food neophobia and picky eating, the role of children's emotional undereating remains unclear. Specifically, we hypothesized that (1) parental food refusal, (2) pressuring feeding practices, and (3) a lack of emotional acceptance of the child would each be associated with higher levels of children's food neophobia and picky eating, and that these associations would be mediated by children's emotional undereating as a maladaptive emotion regulation strategy.

## 2 Methods

### 2.1 Participants

Dutch-speaking parents of children aged 3–8 were recruited in Belgium. The call for participation announced that preferably the parent who was most involved in the feeding of the child would complete the questionnaire. A total of 171 parents completed the questionnaire. One participant was not included in the analyses because her child was already nine years old. Of the 170 parents that were included, 96.5% (*N* = 164) were mothers, and 3.5% (*N* = 6) were fathers. Regarding the sex of the children, 51.8% (*N* = 88) were boys and 48.2% (*N* = 82) were girls. Children's ages ranged from 3 to 8 years old (*M* = 5.45, *SD* = 1.69).

### 2.2 Procedure

Participants were recruited through flyers distributed at elementary schools. Interested parents provided contact information on the flyer, which their child returned to school. Researchers then contacted the parents and provided information on the procedure of the study. If participants agreed to continue, the researcher requested them to complete an active informed consent containing detailed study information and return it by mail. This study was part of a larger project on eating difficulties in young children, in which we also collected interview and observational data. In this study, our focus is solely on describing the questionnaires included in the procedure. Parents received a link and a unique code to complete a secured questionnaire anonymously. This procedure was approved by the Faculty's Ethics Committee (REF 201469).

### 2.3 Instruments

#### 2.3.1 Child food Neophobia

Parents completed the Child Food Neophobia Scale (CFNS) (41), which assesses the extent to which children reject novel or unknown foods. The scale uses a 4-point Likert scale from 1 (strongly disagree) to 4 (strongly agree), with higher scores indicating greater levels of food neophobia. This scale originally consists of 10 items but was adapted for our sample's age range to 6 items (42, 43), includes items such as “My child does not trust new foods” and “My child is afraid to eat things (s)he has never had before”. The CFNS is validated against a behavioral measure of food neophobia (i.e., children's actual willingness to taste when confronted with ten unfamiliar foods) (41), with an internal consistency of *α* = .90 in our sample.

#### 2.3.2 Child Picky eating and emotional undereating

Parents completed the Dutch version of the Child Eating Behavior Questionnaire (CEBQ) (44). The CEBQ is a 35-item parent-report questionnaire that assesses eating-approach as well as eating-avoidance behavior, scored on a 5-point Likert Scale from 1 (never) to 5 (always), with higher scores indicating a stronger display of food approach or food avoidance behavior. Food Avoidance Behavior consists of 4 subscales: Satiety Responsiveness, Food Fussiness, Slowness in Eating, and Emotional Undereating (21). In this study, the subscale Food Fussiness (e.g., “My child likes to eat a large variety of foods”, “It is difficult to please my child with meals”) and the subscale Emotional Undereating was used (e.g., “My child eats less when (s)he is upset”). The questionnaire was found to be a psychometrically sound tool to measure these eating behaviors (44), with an internal consistency of *α* = .88 for Food Fussiness and *α* = .79 for Emotional Undereating.

#### 2.3.3 Parental modeling of food refusal behavior

We assessed parental modeling of food refusal behaviors with four items constructed to measure parents' inclination toward trying new or unfamiliar foods and their preference for a diverse range of foods. The items were scored on a 7-point Likert Scale from 1 (never) to 7 (always). Items were “I like to taste products I have never eaten before”, “I like to taste products I didn't like before”, “I like to eat and drink healthy products” and “I like to eat and drink a large variety of products”. Higher scores on this scale indicate less food refusal behaviors. Internal consistency in the present sample was *α* = .76.

#### 2.3.4 Parental pressure to eat

The Child Feeding Questionnaire (45, 46) is a 31-item questionnaire for parents of children aged 2–11, using a 5-point Likert-scale ranging from 1 (Never) to 5 (always). It includes seven subscales, and in this study, only the “Pressure to Eat” subscale was used, which consists of four items (e.g., “My child always has to empty his/her entire plate”). Higher scores indicate more pressure to eat. The Cronbach's alpha was *α* = .53 in this study^1^.

#### 2.3.5 Parental acceptance of the child

The Nijmeegse Parental Stress Index-short version [NOSI-K; (47)] assesses the levels of stress experienced by parents while parenting children aged 2–14 years. The NOSI-K consists of 25 items, scored on a 6-point Likert-scale ranging from 1 (entirely not agree) to 6 (entirely agree) with higher scores indicating more experienced stress. To operationalize the emotional family climate, participants completed the 3-item subscale “acceptance of the child”: “It is not easy to accept my child as (s)he is”, “my child does things that make me worry a lot” and “my child is sometimes difficult, which makes it not easy to have such a child”. Higher scores reflect less parental acceptance of the child. This questionnaire was found to be reliable and valid (48). Cronbach's alpha in this study was *α* = .78.

### 2.4 Statistical analyses

Analyses were conducted with SPSS version 23. First, missing data were estimated (49). Next, descriptive analyses were conducted for the study variables and Pearson correlations were calculated between age and the study variables. Univariate Analyses of Variance (ANOVA) was performed to investigate sex differences for the different study variables. Following the recommendations by MacKinnon et al. (50), bootstrapping, a non-parametric resampling procedure, was used to test the different mediation models. This bootstrapping procedure was performed using the SPSS macro “mediate” developed by Hayes and Preacher (51). Bootstrapping is superior to previous approaches, such as the procedure of Baron and Kenny (52), as it additionally tests the significance of the indirect effects and is non-sensitive to violations of normality in the data (53). In the current study, 5,000 bootstrap resamples, replacing the original sample (*N* = 170), were used to derive the 95% confidence intervals (CI) for the indirect effects.

The present study was exploratory in nature and sample size was primarily determined by feasibility constraints (i.e., available resources). A formal *a priori* power analysis was therefore not conducted. To evaluate the adequacy of the achieved sample size, a sensitivity power analysis was performed. For correlation and regression analyses (*α* = .05, power = .80), the present sample size (*N* = 170) was sufficient to detect small-to-moderate effects (approximately *r*  ≈ .20–.25 or f^2^ ≈ .05–.08).

For mediation analyses using bootstrapped confidence intervals, previous simulation studies indicate that samples of this magnitude are generally adequate to detect small-to-moderate indirect effects in simple mediation models (54, 55).

## 3 Results

### 3.1 Missing values

Of the total sample (*N* = 170), 140 parents (82%) provided complete data on all the variables of interest. Comparison of means and covariances of all questionnaire variables using Missing Completely At Random (MCAR) test (56) revealed a normed *χ*^2^ of 1.10, *χ*^2^ (325) = 358.59, *p* > .05, indicating that the data were likely missing at random (57). As a consequence, missing values could be estimated, and it was decided to estimate them following the expectation maximization (EM) algorithm available in SPSS (49).

### 3.2 Descriptives and associations between the study variables, age, and gender

Descriptive statistics and associations between the study variables are depicted in Table 1. Results show a significant negative correlation between age and Emotional Undereating (*r* = −.23; *p* = .003), indicating that levels of Emotional Undereating decrease with age. Because of this significant result, we controlled for age in the mediation analyses. No other significant associations were found between age and the other study variables. Furthermore, results of the ANOVAs demonstrate no significant differences between boys and girls on any of the study variables.

**Table 1:** Descriptive statistics and associations between the study variables.

Variable	1	2	3	4	5	6	Boys *M (SD)*	Girls *M (SD)*	*F*
1. Age							5.27 (1.71)	5.65 (1.65)	2.10
2. Modeling	−.03						19.48 (2.26)	19.27 (2.58)	.31
3. Pressure to eat	−.11	−.04					4.07 (.70)	3.96 (.71)	1.07
4. Acceptance	−.00	−.09	−.00				2.78 (.99)	3.04 (1.15)	2.32
5. Emotional Undereating	−.23**	.06	.28***	.19*			15.80 (2.96)	14.93 (3.50)	3.07
6. Food neophobia	.02	−.23**	.33***	.18*	.22**		19.20 (4.84)	19.49 (4.83)	.15
7. Picky Eating	−.07	−.23**	.34***	.14	.22**	.88***	17.98 (4.45)	17.83 (3.99)	.05

* *p* < .05.

** *p* < .01.

*** *p* < .001.

Higher scores on Modeling reflect less modeling of food refusal behaviors; Higher scores on Acceptance reflect less acceptance of the child; ANOVAs demonstrate no significant gender differences (all *p*-values > .05).

Pressure to Eat was significantly correlated with Emotional Undereating (*r* = .28; *p* < .001), Food Neophobia (*r* = .33; *p* < .001) and Picky Eating (*r* = .34; *p* < .001). Acceptance was also significantly correlated with Emotional Undereating (*r* = .19; *p* = .015) and Food Neophobia (*r* = .18; *p* = .018). Modeling was significantly correlated with Food Neophobia (*r* = -.23; *p* = .002) and Picky Eating (*r* = -.23; *p* = .002). Emotional Undereating was also significantly correlated with Food Neophobia (*r* = .22; *p* = .005) and Picky Eating (*r* = .22; *p* = .005). Finally, Food Neophobia and Picky Eating were significantly correlated as well (*r* = .88; *p* < .001).

### 3.3 Does emotional undereating mediate the association between modeling and food Neophobia/Picky eating?

**Figure 1:**
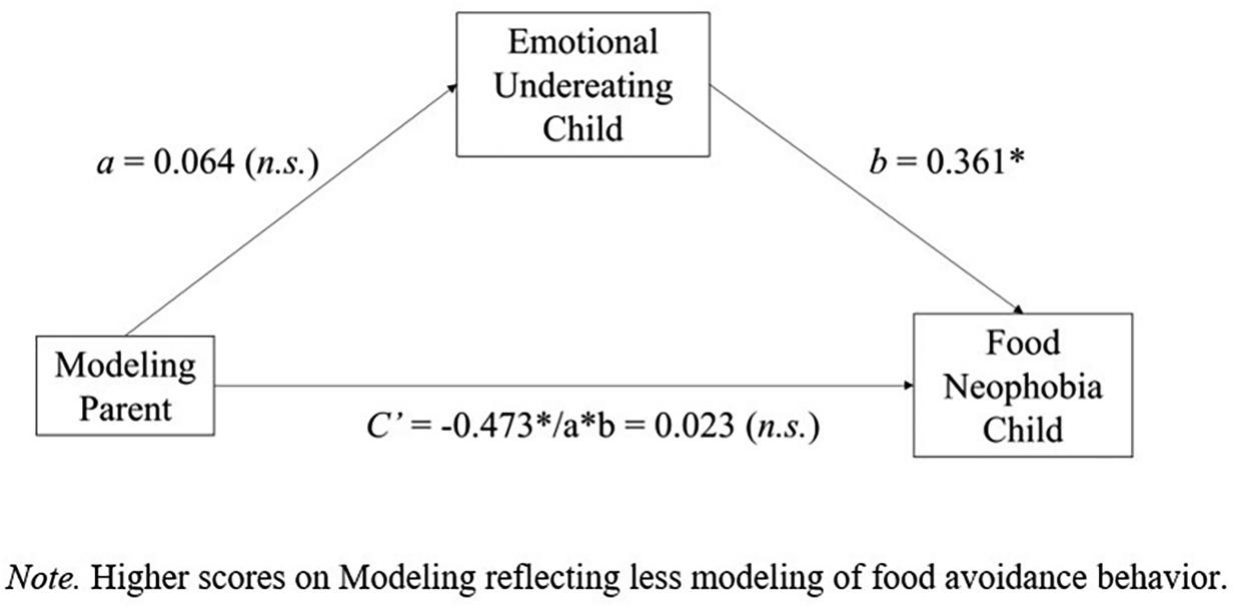
Associations between modeling, emotional undereating and food neophobia in 3 to 8-year-old children.

Results (see also Figure 1 and Table 2) show a significant direct effect of Modeling on Food Neophobia, *B* = −0.473, *SE* = 0.147, *p* = .002, 95% CI [−0.763, −0.184], indicating that higher levels of Modeling of food refusal behaviors by the parent (i.e., lower scores on Modeling) were associated with higher levels of Food Neophobia in the child. However, no significant effect was found of Modeling on Emotional Undereating, *B* = 0.064, *SE* = 0.101, *p* = .526, 95% CI [−0.136, 0.265]. Results did show a significant positive effect of Emotional Undereating on Food Neophobia, *B* = 0.361, *SE* = 0.112, *p* = .002, 95% CI [0.140, 0.581]. The indirect effect of Modeling on Food Neophobia via Emotional Undereating was not significant, *B* = 0.023, boot*SE* = 0.040, 95% CI [−0.062, 0.101].

**Table 2:** Overview of the results of the mediation analyses.

	*Estimate*	*SE*	*t*	*p*	*95% CI*
Paths to (a)/from mediator (b)					
Modeling → EU	0.064	0.101	0.636	.526	[−0.136, 0.265]
Pressure → EU	1.173	0.339	3.464	.001*	[0.504, 1.841]
Acceptance → EU	0.564	0.224	2.514	.013*	[0.121, 1.007]
EU → Food Neophobia	0.361	0.112	3.225	.002*	[0.140, 0.581]
EU → Picky Eating	0.287	0.098	2.935	.004*	[0.094, 0.481]
Indirect effects (a*b)					
Modeling → EU → Food Neophobia	0.023	0.040	/	/	[−0.062, 0.101]
Pressure → EU → Food Neophobia	0.266	0.182	/	/	[−0.006, 0.694]
Acceptance → EU → Food Neophobia	0.170	0.099	/	/	[0.013, 0.400]*
Modeling → EU → Picky Eating	0.019	0.032	/	/	[−0.050, 0.083]
Pressure → EU → Picky Eating	0.194	0.155	/	/	[−0.044, 0.568]
Acceptance → EU → Picky Eating	0.137	0.084	/	/	[0.005, 0.326]*
Direct effects (c’)					
Modeling → Food Neophobia	−0.473	0.147	−3.223	.002*	[−0.763, −0.184]
Pressure → Food Neophobia	2.024	0.516	3.919	.000*	[1.004, 3.043]
Acceptance →Food Neophobia	0.649	0.343	1.893	.060	[−0.028, 1.325]
Modeling → Picky Eating	−0.428	0.128	−3.334	.001*	[−0.682, −0.175]
Pressure → Picky Eating	1.848	0.452	4.093	.000*	[0.957, 2.740]
Acceptance → Picky Eating	0.431	0.302	1.425	.156	[−0.166, 1.027]
Total effects (c = c’ + a*b)					
Modeling → Food Neophobia	−0.450	0.151	−2.990	.003*	[−0.747, −0.153]
Pressure → Food Neophobia	2.290	0.503	4.551	.000*	[1.297, 3.283]
Acceptance →Food Neophobia	0.818	0.342	2.392	.018*	[0.143, 1.493]
Modeling → Picky Eating	−0.410	0.131	−3.123	.002*	[−0.669, −0.151]
Pressure → Picky Eating	2.042	0.439	4.658	.000*	[1.177, 2.908]
Acceptance → Picky Eating	0.568	0.301	1.888	.061	[−0.026, 1.162]

*Note*. EU, Emotional Undereating; all mediation analyses controlled for age. Higher scores on Modeling reflect less modeling of food refusal behavior; Higher scores on Acceptance reflect less acceptance of the child. Exact (uncorrected) *p*-values are reported. A false discovery rate (FDR) correction was applied for multiple testing (adjusted *α* = .0375). .

* Effects remaining significant after FDR correction are marked with an asterisk (*).

**Figure 2:**
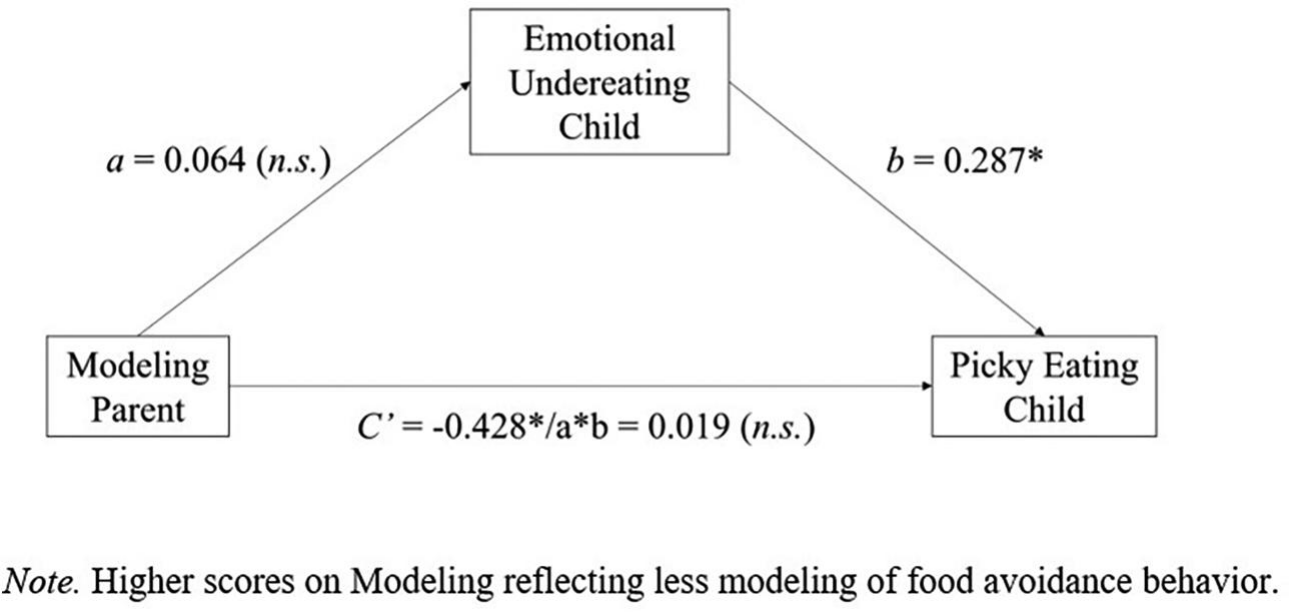
Associations between modeling, emotional undereating and picky eating in 3 to 8-year-old children.

Results (see also Figure 2 and Table 2) also show a significant direct effect of Modeling on Picky Eating, *B* = −0.428, *SE* = 0.128, *p* = .001, 95% CI [−0.682, −0.175] indicating that higher levels of Modeling of food refusal behaviors by the parent (i.e., lower scores on Modeling) were associated with higher levels of Picky Eating in the child. Again, no significant effect was found of Modeling on Emotional Undereating, *B* = 0.064, *SE* = 0.101, *p* = .526, 95% CI [−0.136, 0.265]. Results did show a significant positive effect of Emotional Undereating on Picky Eating, *B* = 0.287, *SE* = 0.098, *p* = .004, 95% CI [0.094, 0.481]. The indirect effect of Modeling on Picky Eating via Emotional Undereating was not significant, *B* = 0.019, boot*SE* = 0.032, 95% CI [−0.050, 0.083].

### 3.4 Does emotional undereating mediate the association between pressure to eat and food Neophobia/Picky eating?

**Figure 3:**
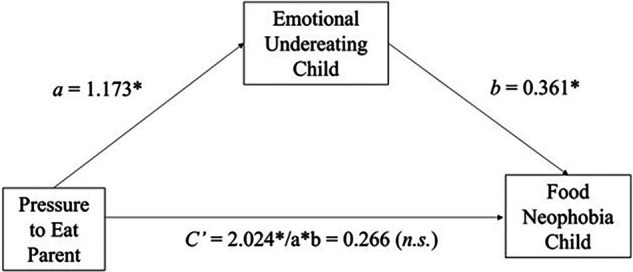
Associations between pressure to Eat, emotional undereating and food neophobia in 3 to 8-year-old children.

Results (see also Figure 3 and Table 2) demonstrate a significant direct effect of Pressure to Eat on Food Neophobia, *B* = 2.024, *SE* = 0.516, *p* < .001, 95% CI [1.004, 3.043] indicating that higher levels of Pressure to Eat by the parent were associated with higher levels of Food Neophobia in the child. Also, a significant positive effect was found of Pressure to Eat on Emotional Undereating, *B* = 1.173, *SE* = .339, *p* = .001, 95% CI [0.504, 1.841]. Results also show a significant positive effect of Emotional Undereating on Food Neophobia, *B* = 0.23, *SE* = 0.11, *p* = .048, 95% CI [0.002, 0.452]. The indirect effect of Pressure to Eat on Food Neophobia via Emotional Undereating was however not significant, *B* = 0.266, boot*SE* = 0.182, 95% CI [−0.006, 0.694].

**Figure 4:**
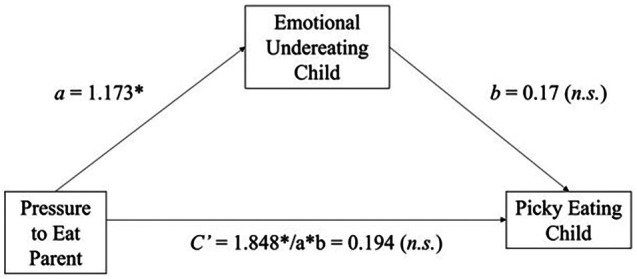
Associations between pressure to Eat, emotional undereating and picky eating in 3 to 8-year-old children.

Results also demonstrate a significant direct effect of Pressure to Eat on Picky Eating, *B* = 1.848, *SE* = 0.452, *p* = .000, 95% CI [0.957, 2.740] indicating that higher levels of Pressure to Eat by the parent were associated with higher levels of Picky Eating in the child (see also Figure 4 and Table 2). Again, a significant positive effect was found of Pressure to Eat on Emotional Undereating, *B* = 1.173, *SE* = .339, *p* = .001, 95% CI [0.504, 1.841]. Results show no significant effect of Emotional Undereating on Picky Eating, *B* = 0.17, *SE* = 0.10, *p* = .10, 95% CI [−0.031, 0.363]. The indirect effect of Pressure to Eat on Picky Eating via Emotional Undereating was not significant, *B* = 0.194, boot*SE* = 0.155, 95% CI [−0.044, 0.568].

### 3.5 Does emotional undereating mediate the association between acceptance and Food Neophobia?

**Figure 5:**
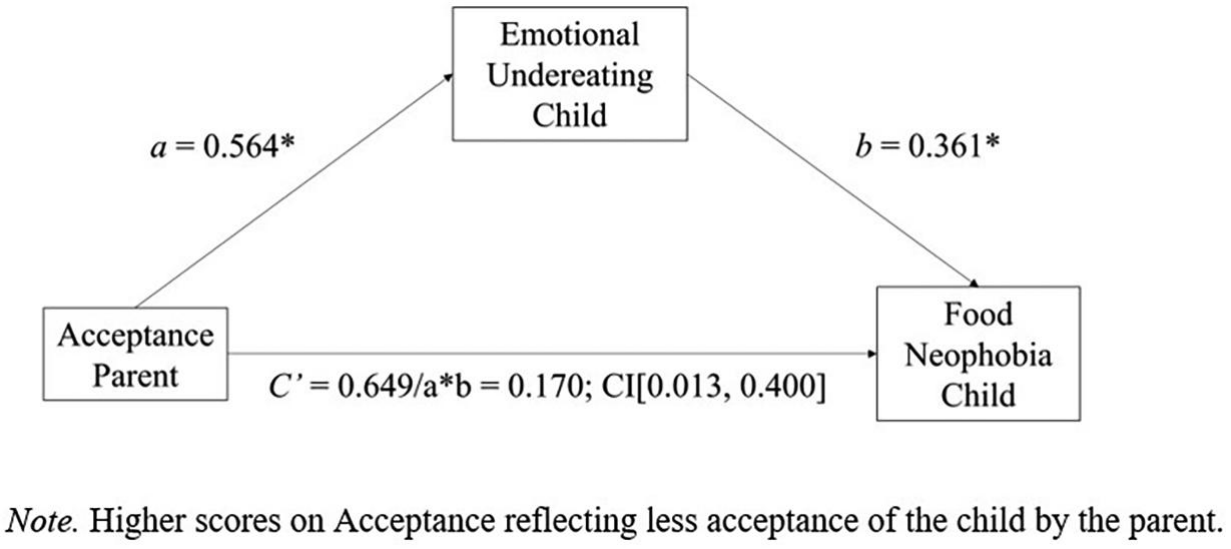
Associations between acceptance, emotional undereating and food Neophobia in 3 to 8-year-old children.

Results (see also Figure 5 and Table 2) demonstrate a trend significant direct effect of Acceptance on Food Neophobia, *B* = 0.649, *SE* = 0.343, *p* = .060, 95% CI [−0.028, 1.325] indicating that less acceptance of the child by the parent (reflected in higher scores on the Acceptance variable) was associated with higher levels of Food Neophobia in the child. Also, a significant effect was found of Acceptance on Emotional Undereating, *B* = 0.564, *SE* = 0.224, *p* = .013, 95% CI [0.121, 1.007] indicating that less acceptance of the child by the parent (reflected in higher scores on the Acceptance variable) was associated with more Emotional Undereating in the child. Results also show a significant effect of Emotional Undereating on Food Neophobia, *B* = 0.30, *SE* = 0.12, *p* = .01, 95% CI [0.072, 0.530]. The indirect effect of Acceptance on Food Neophobia via Emotional Undereating was also significant, *B* = 0.170, boot*SE* = 0.099, 95% CI [0.013, 0.400], thereby showing evidence for the proposed mediation model. More specifically, 20.7% of the total effect of Acceptance on Food Neophobia seems to operate indirectly through Emotional Undereating.

**Figure 6:**
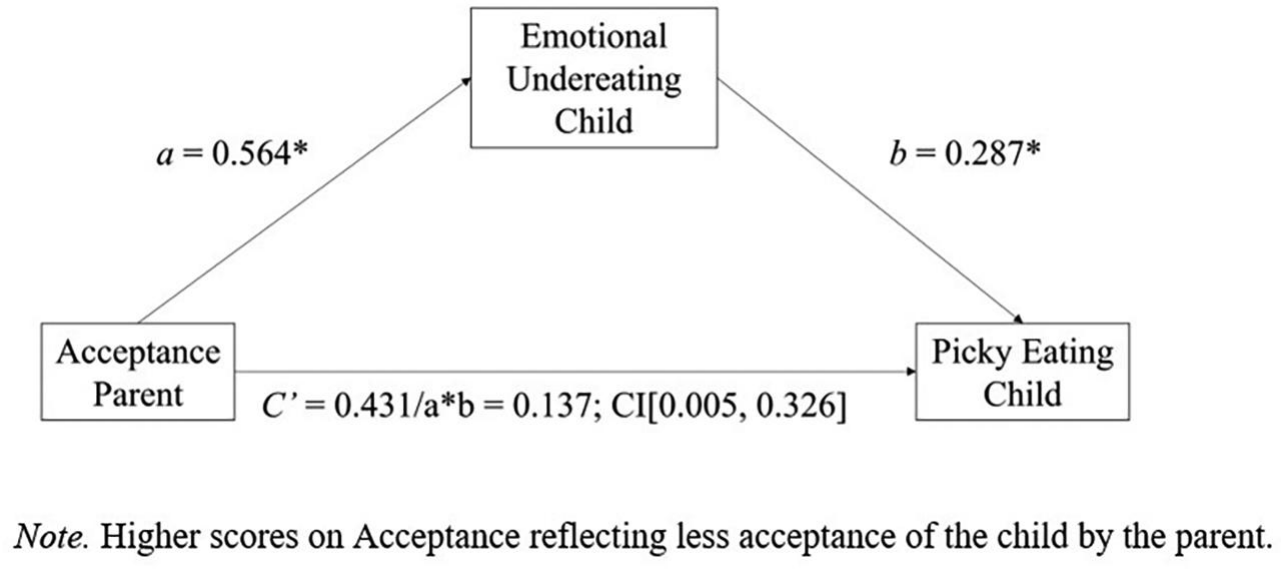
Associations between acceptance, emotional undereating and Picky eating in 3 to 8-year-old children.

Results (see also Figure 6 and Table 2) demonstrate no significant direct effect of Acceptance on Picky Eating, *B* = 0.431, *SE* = 0.302, *p* = .156, 95% CI [−0.166, 1.027]. Again, a significant effect was found of Acceptance on Emotional Undereating, *B* = 0.564, *SE* = 0.224, *p* = .013, 95% CI [0.121, 1.007]. Results also show a significant effect of Emotional Undereating on Picky Eating, *B* = 0.24, *SE* = 0.10, *p* = .02, 95% CI [0.042, 0.446]. The indirect effect of Acceptance on Picky Eating via Emotional Undereating was also significant, *B* = 0.137, boot*SE* = 0.084, 95% CI [0.005, 0.326], thereby showing evidence for the proposed mediation model. More specifically, 24.6% of the total effect of Acceptance on Picky Eating seems to operate indirectly through Emotional Undereating.

## 4 Discussion

We investigated how family context variables and children's emotion regulation are related to children's food neophobia and picky eating. In line with our hypotheses, we found that emotional undereating mediated the relationship between a lack of parental acceptance and both food neophobia and picky eating in children. This suggests that children may use reduced eating as a coping strategy when confronted with negative emotions. Although previous research has linked negative emotionality to food refusal (58, 59), the role of emotional undereating in explaining these types of behavior has been less explored. Our results show that inadequate emotion regulation strategies in young children are related to their eating behaviors.

Additionally, emotional undereating is associated with the family's emotional climate, specifically the child's acceptance by their caregiver. This aligns with recent longitudinal findings that poor family functioning increases the risk of emotional undereating in children (20). Our mediation analyses show that 20.7% of the association between parental acceptance and food neophobia, and 24.6% of the association between parental acceptance and picky eating, were mediated by emotional undereating. This suggests that lower parental acceptance may prevent children from feeling secure, leading them to adopt avoidant emotion regulation strategies (37, 60), potentially influencing their eating behavior. Reduced parental acceptance might also exacerbate food avoidance, increasing parental stress and potentially teaching maladaptive avoidance strategies. Previous studies have linked insecure attachment to eating disorder symptoms in children and adolescents (40, 61). Furthermore, a systematic review and meta-analysis demonstrated that insecure attachment is related to both clinical and subclinical eating disorder symptoms, with maladaptive emotion regulation as one of the strongest mediators underlying this relationship (39). The current study is the first to show this indirect association in the context of food neophobia. Furthermore, maladaptive emotion regulation appears relevant to both food neophobia and picky eating, but further research is needed to replicate these findings.

Emotional undereating did not mediate the relationship between parental modeling of food neophobic behaviors and the outcome variables, contrary to the first pathway proposed by the Tripartite Model proposed by Morris et al. (28). One possible explanation might be how our independent variable, modeling, was operationalized. Specifically, modeling was operationalized through the assessment of parents' own tendencies to try new or varied foods. Instead of operating via emotional undereating, these tendencies may be modeled more directly, as demonstrated by the significant direct effects between parent's own tendencies to try new or varied foods and children's food neophobia and picky eating in our study, which is also consistent with previous research (29, 32). This interpretation is consistent with the Tripartite Model proposed by Morris et al. (28), which proposes not only indirect pathways through emotional regulation, but also a direct pathway through observational learning. Children may acquire eating-related behaviors by observing their parents' reactions in food-related situations. For example, when parents consistently avoid unfamiliar foods or exhibit anxiety toward novel foods, children may internalize similar behaviors and attitudes. Such observational learning mechanisms have also been observed in studies examining other eating problems and disorders [e.g., (62)]. To further investigate this modeling pathway, future research should explore parents' emotional undereating or other emotion regulation strategies and observe both parents' and children's emotional responses to novel foods.

Contrary to our hypotheses, emotional undereating does not mediate the association between pressuring the child to eat and the two outcome variables. Consistent with previous research on parental feeding practices and feeding problems (32, 63) we found a significant direct association between pressure to eat and both food neophobia and picky eating. We also observed a significant association between pressure to eat and emotional undereating. This finding suggests that this feeding practice may not only be employed by parents to control children's eating behavior, but may also unintentionally disrupt children's internal hunger and satiety cues or induce negative affect. Morris et al. (28) suggest that an optimal level of control exists where parents allow the expression of negative emotions without overly encouraging it. Future research should explore this hypothesis further. While we focused on pressure to eat as a specific controlling practice, other related parenting practices, such as emotional coaching and teaching emotion regulation strategies, may better explain children's emotional undereating and food refusal behaviors.

### 4.1 Strengths, limitations and directions for future research

This study has several strengths. First, it examined both food neophobia and picky eating, which are behaviorally distinct and may be explained by different factors (3). This allowed us to examine whether different or identical pathways explain these types of behavior. Our hypotheses were grounded in the Tripartite Model of familial influence on emotion regulation (28), marking the first study to also examine the mediating role of emotion regulation. Additionally, we recruited a diverse sample of parents of boys and girls aged three to eight years.

There are also several limitations that need to be acknowledged. First, the design of this study was cross-sectional and therefore, it was not possible to draw any conclusions about the directionality of effects. Future research should longitudinally examine the proposed pathways. Second, as the current sample was recruited from the general population, results cannot be generalized to children with clinical feeding or eating disorders. Future research should investigate whether these pathways explaining food refusal behaviors in the general community are also present in clinical samples. Third, we used self-report questionnaires with the parents as informants in the present study, which may increase a mono-method bias. Therefore, future studies may include multiple informants (e.g., children and parents), as well as multiple methods to examine the proposed hypotheses. Clinical interviews, such as the Pica, ARFID, and Rumination Disorder Interview [PARDI; (64)] could provide a more refined assessment of food refusal behavior, while observational studies in home environments could capture both parents' and children's emotional reactions during mealtimes. Furthermore, wearable technologies may provide objective measures of emotional and physiological reactions to food-related sensory stimuli. Finally, advanced analytic approaches, including artificial intelligence techniques may help disentangle complex indirect relationships between clinical, emotional, and food-related features. Fourth, as the Cronbach's alpha of the CFQ's pressure to eat scale was low, results using this scale should be interpreted with caution. Finally, as our call for participation announced that preferably the parent who was most involved in the feeding of the child would complete the questionnaire, our data demonstrate that, in most families, the mother completed the questionnaire. Although this was not unexpected, our conclusions regarding the role of family context variables may be specifically applicable for mothers and thus not necessarily be generalizable to fathers. Recent evidence however also points to an important role of fathers and dynamics between both parents in explaining picky eating (65). The number of participating fathers in the present study was too small to allow for separate analyses, but future research may include both mothers and fathers to further investigate the role of the family context in food refusal behaviors. Also, apart from including other variables and actors to operationalize the family context, as well as more intrapersonal factors (e.g., child temperament), future research may also consider the (mediating) role of other deactivating and hyperactivating maladaptive emotion regulation strategies such as suppression and rumination (61, 66).

### 4.2 Theoretical and clinical implications

Our findings contribute to theories explaining food refusal in children and highlight the impact of parent-child relationships on disordered eating [e.g., (40)]. Clinically, this underscores the need to consider family context variables and emotion regulation in the assessment and treatment of child food refusal behaviors. Interventions should not only address eating behavior but also teach adaptive strategies for dealing with novel or unfamiliar foods and managing emotions such as anxiety or disgust. These insights may also guide the development of ARFID prevention programs that focus on improving family dynamics and helping families manage emotions related to eating and food avoidance.

## Data Availability

The original contributions presented in the study are included in the article/Supplementary Material, further inquiries can be directed to the corresponding author.

## Footnotes

^1^Removing one item from this subscale resulted in a somewhat higher Cronbach's Alpha of .71. Using this reduced scale did not yield any notably different results and did not affect our conclusions. For this reason we report the results obtained using the original full four-item scale.
